# Identification of Circulating lncRNA Expression Profiles in Patients with Atrial Fibrillation

**DOI:** 10.1155/2020/8872142

**Published:** 2020-11-22

**Authors:** Zhong-bao Ruan, Fei Wang, Bing-di Gongben, Ge-cai Chen, Li Zhu

**Affiliations:** Department of Cardiology, Jiangsu Taizhou People's Hospital, Taizhou 225300, China

## Abstract

**Purpose:**

To investigate the expression profiles of long noncoding RNAs (lncRNAs) in patients with atrial fibrillation (AF).

**Methods:**

The peripheral blood monocytes of a total of 20 patients with AF and 20 healthy subjects were collected for gene chip technology to detect differentially expressed lncRNAs from 2017.01 to 2017.08. Reverse transcription polymerase chain reaction (RT-PCR) was applied for further verification. Gene Ontology (GO) and Kyoto Encyclopedia of Genes and Genomes (KEGG) analyses were performed to identify the functions of differentially expressed genes and related pathways.

**Results:**

There were 19 lncRNAs differentially expressed (FC ≥ 2, *P* < 0.05), of which 6 were upregulated and 13 were downregulated. Two of three upregulated lncRNAs (*P* = 0.014 and 0.006 for HNRNPU-AS1 and LINC00861, respectively) and two of three downregulated lncRNAs (*P* = 0.028 and 0.032 for RP11-443B7.3 and CTD-2616J11.14, respectively) were randomly confirmed by RT-PCR and showed a significantly different expression with the RNA-seq results. GO analysis showed that differentially expressed genes enriched in differentially expressed transcripts in biological process were mainly involved in metabolic process, catabolic process, and biosynthetic process. Differentially expressed transcripts in cellular component were mainly involved in nuclear lumen, organelle lumen, and cytoplasm. Differentially expressed transcripts in molecular function were mainly involved in protein binding, RNA binding, and molecular function. KEGG enrichment pathway analysis showed that some of the enrichment pathways associated with differentially expressed lncRNAs include calcium signaling pathway, NF-kappa B signaling pathway, cytokine-cytokine receptor interaction, and Toll-like receptor signaling pathway. HNRNPU-AS1 was the highest positive correlated lncRNA in the networks.

**Conclusions:**

The expression of lncRNA in peripheral blood of AF patients is different from that of normal people. The physiological functions of these differentially expressed lncRNAs may be related to the pathogenesis of AF, which provide experimental basis and new therapeutic target for prognosis and treatment of patients with AF. HNRNPU-AS1 may play an important role in the pathophysiology and mechanisms of AF.

## 1. Introduction

Atrial fibrillation (AF) is one of the most common cardiac arrhythmias. There is a positive relation between the prevalence of AF and age, with a prevalence of 0.5% among individuals aged 50 to 59 years and up to 8.8% among individuals aged 80 to 89 years [1]. As reported, AF is associated with increased global public health risk such as stroke, heart failure, and cardiovascular mortality [2, 3]. Over the last 20 years, the major mechanisms involved in AF have been illustrated as electrical and structural remodeling, reentry and ectopic firing, inflammation, and oxidative stress [4, 5]. However, the exact underlying pathophysiology and mechanisms about AF remain unclear.

Long noncoding RNAs (lncRNAs) are non-protein-coding RNAs longer than 200 nucleotides in length and seldom encode proteins [6]. Accumulating studies have demonstrated that lncRNAs play a vital role in the regulation of almost all biological processes, including development, differentiation, and metabolism. Moreover, lots of lncRNAs have been functionally elucidated in regulating a great number of genes involved in cancers, myocardial infarction, and heart failure and may be potential novel biomarkers for disease diagnosis and prognosis, as well as therapeutic targets [7–9]. However, relationships between AF and lncRNAs are still unknown. Recently, we have performed microarray analyses on human left atrial appendage in AF patients and indicated that there are differentially expressed lncRNAs in AF, which may contribute to the pathogenesis of AF [10].

As a type of white blood cell present in the peripheral circulation, monocytes have been reported to be closely linked to outcomes in patients with cardiovascular disease and the high numbers of monocytes are associated with increased risk of recurrent myocardial infarction, hospitalization, and cardiac death [11, 12]. Yet, the role of monocytes in determining outcomes among AF patients is unknown; the lncRNA expression of peripheral circulating monocytes in AF has not been investigated. Thus, in the present study, in order to determine whether there is a dysregulation of lncRNAs in monocytes of AF patients compared with healthy people, lncRNA microarray is used and real-time quantitative reverse transcription PCR (qRT-PCR) for specific differentially expressed lncRNAs is used subsequently to validate the microarray results. We think that these data could help highlight the exact underlying pathophysiology and mechanisms about AF and provide new therapeutic strategies in AF patients.

## 2. Materials and Methods

### 2.1. Study Population

In this study, 20 patients with AF and 20 matched healthy control subjects were recruited from our hospital. Written informed consent was obtained from AF patients and controls before entering this experiment, and the study protocol was approved by the Ethics Committee of Jiangsu Taizhou People's Hospital. The diagnosis of AF was mainly based on the criteria listed in 2016 ESC guidelines for the management of atrial fibrillation [13]. Patients were excluded if they were combined with hyperthyroidism, chronic pulmonary heart disease, valvular heart disease, previous coronary atherosclerotic heart disease, infective endocarditis, severe dysfunction of the liver and kidney, autoimmune disease, and malignant tumors. Healthy control subjects were the population without AF and the diseases mentioned above. Information about baseline characteristics was collected from the medical records by a special doctor and provided in Table 1.

### 2.2. Monocyte Collection, RNA Preparation, and Construction of the lncRNA Microarray

About 8 ml of blood samples from each subject was drawn into ethylenediaminetetraacetic acid- (EDTA-) anticoagulant tubes, diluted 1 : 1 with PBS, separated with Ficoll's density gradient centrifugation, and underwent subsequent magnetic cell sorting. Total RNAs were isolated from the isolated monocytes using TRIzol (Invitrogen, USA) and were quantified using a NanoDrop spectrophotometer (IMPLEN, CA, USA). A total amount of 3 *μ*g RNA per sample was used as input material for the RNA sample preparations, products were purified (AMPure XP system), and library quality was assessed on the Agilent Bioanalyzer 2100 system. Quantile normalization and subsequent data processing were performed using the GeneSpring GX v11.5.1 software package (Agilent, USA). Differentially expressed lncRNAs between the two groups were identified by fold change filtering. The threshold set for upregulated and downregulated lncRNAs was more than twofold.

### 2.3. Validation by Reverse Transcription Polymerase Chain Reaction (RT-PCR)

To verify the results of microarray data, 6 differentially expressed lncRNAs were selected randomly for RT-PCR, including 3 upregulated lncRNAs (HNRNPU-AS1, AC005786.7, and LINC00861) and 3 downregulated circRNAs (RP11-443B7.3, CTD-2616J11.14, and CTD-2616J11.3). Briefly, a total of 3 *μ*g RNA was used for reverse transcription. Detection of the amplified cDNA was performed with the rotor gene Q series (Qiagen, USA). The GeneAmp PCR System 7500 (Applied Biosystems, USA) was used for RT-PCR. 1 *μ*l of cDNAs was added to 12.5 *μ*l of SYBR Green Gene Expression Master Mix (Applied Biosystems, Inc.), 10.5 *μ*l of DEPC-treated water, and 0.5 *μ*l of primers. The relative expression levels of the genes were presented as fold changes and normalized to the housekeeping gene GAPDH. The results were analyzed according to the 2^−*ΔΔ*Ct^ method [14]. Primers used for RT-PCR are listed in Table 2.

### 2.4. Gene Ontology (GO) and KEGG Enrichment Analysis

Gene Ontology (GO) enrichment analysis of differentially expressed genes or lncRNA target genes was implemented by the GOseq R package, in which gene length bias was corrected. GO terms with corrected *P* value less than 0.05 were considered significantly enriched by differentially expressed genes. KEGG is a database resource for understanding high-level functions and utilities of the biological system, such as the cell, the organism, and the ecosystem, from molecular-level information, especially large-scale molecular datasets generated by genome sequencing and other high-throughput experimental technologies (http://www.genome.jp/kegg/). We used KOBAS software to test the statistical enrichment of differential expression genes or lncRNA target genes in KEGG pathways.

### 2.5. Construction of lncRNA-mRNA Regulatory Coexpression Network

To evaluate the interaction between the differentially expressed lncRNAs and their target mRNAs, a coexpression network was constructed on the basis of the correlation between the differentially expressed lncRNAs and mRNAs (Pearson correlation coefficient, absolutevalue > 0.95; *P* value < 0.05) using the Cytoscape 3.7.0 software (http://cytoscape.org/).

### 2.6. Statistical Analysis

SPSS software 22.0 was used for statistical analyses. Continuous data were presented as mean ± standarddeviation (SD) and analyzed using Student's *t*-test. Categoric variables were expressed as number and analyzed with the chi-square test. Statistical significance was determined at *P* < 0.05. Fisher's exact test was performed in enrichment analysis; *P* < 0.05 and FDR < 0.05 were considered statistically significant. Pearson correlation coefficient was performed to determine the gene coexpression; the absolutevalue > 0.95and *P* value < 0.05 were considered to be statistically correlated between lncRNAs and mRNAs.

## 3. Results

### 3.1. Analysis of Differentially Expressed lncRNAs

**Figure 1:**
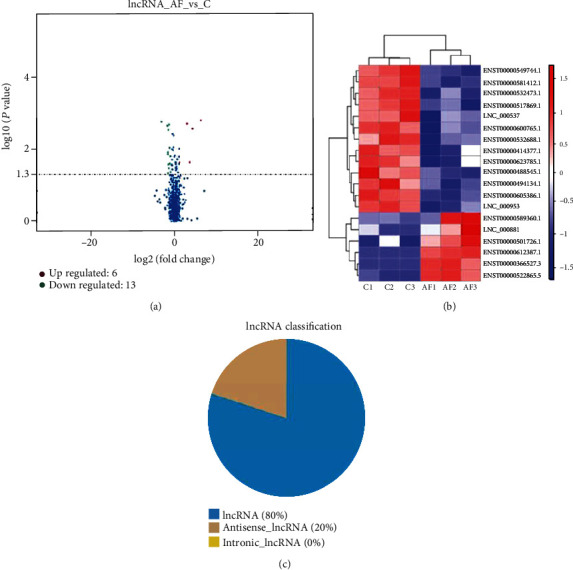
lncRNA microarray expression data between the AF group and the control group. (a) Volcano plot of lncRNAs between the AF group and controls. The horizontal dashed line delimits 2.0-fold up- and downregulation. Red plots represent upregulated lncRNAs, and green plots represent downregulated lncRNAs with >2.0 fold change and corrected *P* value < 0.05. (b) Cluster analysis of differentially expressed lncRNAs of AF patients and healthy controls. In the color scheme, red indicates higher expression, and green indicates lower expression. (c) Category of differentially expressed lncRNAs.

In total, 675 lncRNAs were analyzed by microarray. A total of 19 lncRNAs was calculated as differentially expressed between the AF group and the control group (foldchange > 2 and *P* < 0.05), in which 6 lncRNAs were upregulated and 13 lncRNAs were downregulated (Figure 1 and Table 3).

### 3.2. Microarray Validation by RT-PCR

**Figure 2:**
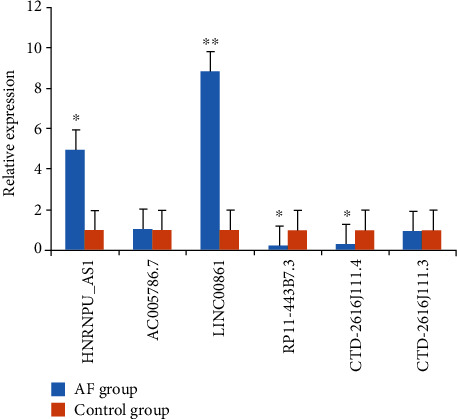
lncRNA expression validated by real-time RT-PCR. Each RT-PCR assay was performed for three times. ^∗^*P* < 0.05 and ^∗∗^*P* < 0.01, compared with the control group.

To validate the microarray results, 6 differentially expressed lncRNAs were randomly selected to verify their expression level by qPCR, including 3 upregulated lncRNAs (HNRNPU-AS1, AC005786.7, and LINC00861) and 3 downregulated circRNAs (RP11-443B7.3, CTD-2616J11.14, and CTD-2616J11.3). As a result, 2 out of 3 upregulated lncRNAs (*P* = 0.014 and 0.006 for HNRNPU-AS1 and LINC00861, respectively) and 2 out of the three downregulated lncRNAs (*P* = 0.028 and 0.032 for RP11-443B7.3 and CTD-2616J11.14, respectively) showed a significantly different expression with the RNA-seq results (Figure 2).

### 3.3. GO and KEGG Pathway Analysis of Differentially Expressed lncRNAs

**Figure 3:**
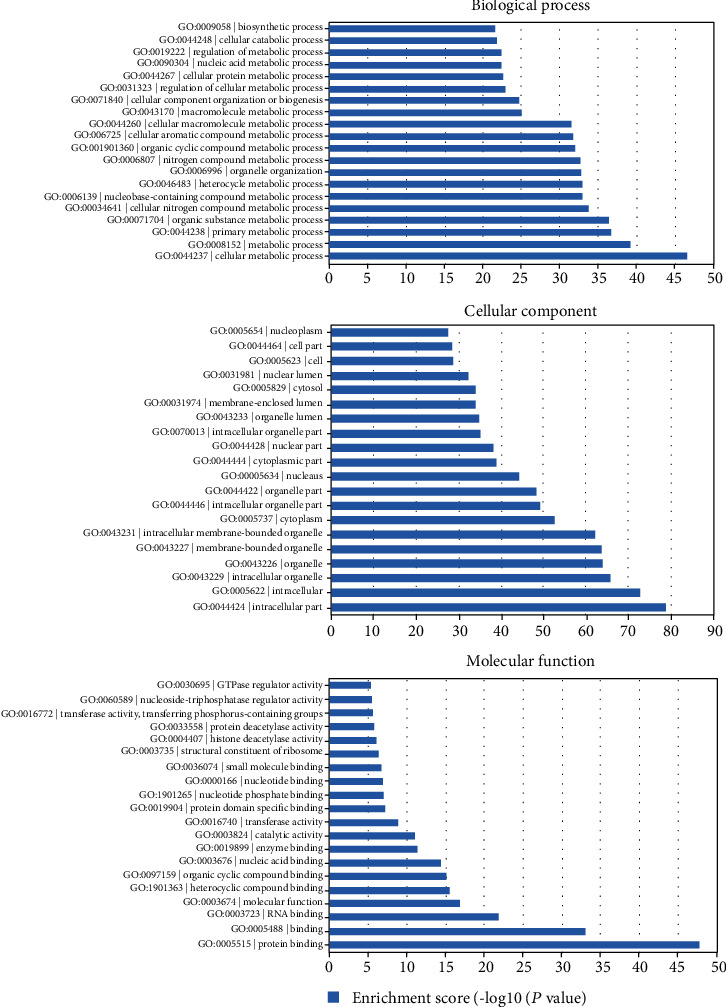
The top 20 results of GO analysis (biological process, cellular component, and molecular function).

**Figure 4:**
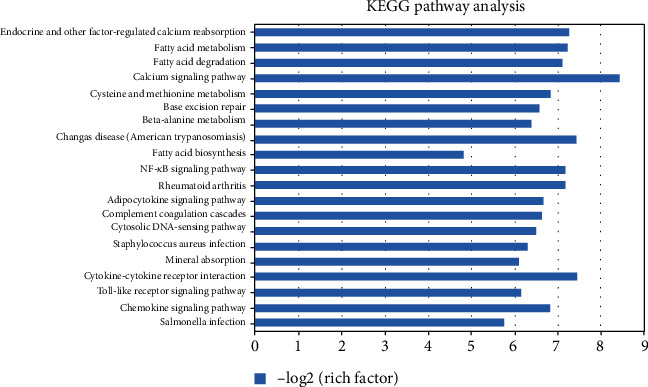
The top 20 neighbor coding genes of KEGG enrichment correspond to the differentially expressed lncRNAs.

Differentially expressed target genes of the lncRNAs were further analyzed by GO analysis, which included biological process, cell component, and molecular function three categories. The top 20 significant GO terms of each subgroup are shown in Figure 3. As shown in Figure 3, differentially expressed transcripts in biological process were mainly involved in metabolic process, catabolic process, and biosynthetic process. Differentially expressed transcripts in cellular component were mainly involved in nuclear lumen, organelle lumen, and cytoplasm. Differentially expressed transcripts in molecular function were mainly involved in protein binding, RNA binding, and molecular function. Next, KEGG pathway enrichment analysis for the differentially expressed lncRNAs was performed to identify pathways and further study biological function. We performed the critical pathways with low *P* values (*P* < 0.05) and used log *P* value to describe the significance level of the pathway enrichment. The top 20 pathway terms of KEGG analysis are shown in Figure 4. Briefly, most enriched pathways were related to the calcium signaling pathway, NF-kappa B signaling pathway, cytokine-cytokine receptor interaction, and Toll-like receptor signaling pathway.

### 3.4. Construction of lncRNA-mRNA Regulatory Coexpression Network

**Figure 5:**
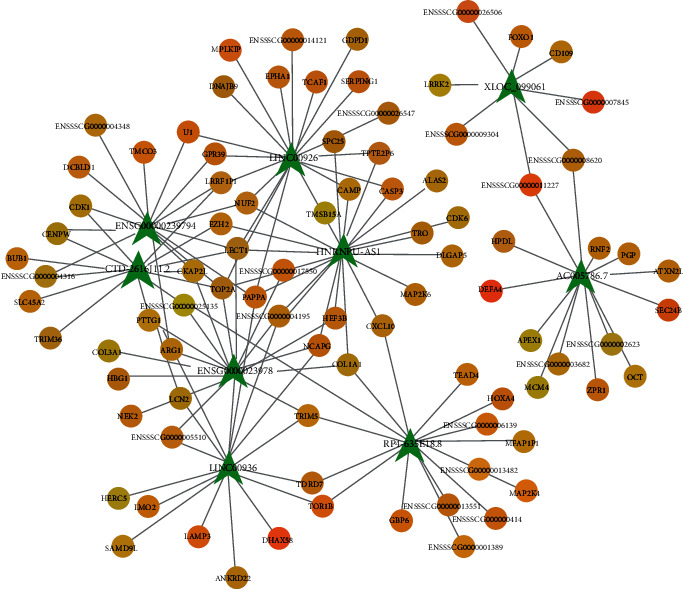
lncRNA-mRNA coexpression network explored by using Cytoscape. The size of each node represents functional connectivity of each lncRNA. The green node represents lncRNA, and the yellow node represents mRNA.

According to the differential expression of lncRNAs, the potential coexpression network between lncRNAs and mRNAs was constructed by using Cytoscape. As shown in Figure 5, HNRNPU-AS1 was the highest positive correlated lncRNA in the networks, which suggests that HNRNPU-AS1 may play an important role in the pathophysiology and mechanisms of AF.

## 4. Discussion

As one of the most common cardiac arrhythmias, AF is responsible for the risk of ischemic stroke and further results in significant morbidity, mortality, and poor quality of life. Various evidence suggests that AF presence affects an estimated 33.5 million individuals in the global world with an overall prevalence of 1-2% in the general population and an increase with age up to 20% in octogenarians [3, 15]. However, the efficacy of presently available therapeutic approaches is limited. Thus, elucidating the mechanisms underlying AF onset and progression is helpful for AF therapeutic innovation. Our previous study has demonstrated that there were differentially expressed lncRNAs in atrial tissues from AF patients and dysregulated lncRNAs may play regulatory roles in the mechanism of AF [10]. To our knowledge, there are very few studies revealing lncRNA expression profiles of monocytes from the AF population. Furthermore, compared with atrial tissues, monocytes are easily obtained and their RNA quality meets the standard of RNA sequencing (RNA-seq).

In the current study, as an outstanding technology for disease excavation, RNA-seq was applied to identify differentially expressed lncRNAs of monocytes between AF patients and healthy people. We identified 19 differentially expressed lncRNAs of monocytes between AF patients and healthy people, among which 6 lncRNAs were downregulated and 13 lncRNAs were upregulated. Compared with the differentially expressed lncRNAs in human left atrial appendage of AF patients, the numbers of differentially expressed lncRNAs in circulating peripheral blood were significantly lower.

To further identify whether the differentially expressed lncRNAs are associated with AF, 3 upregulated lncRNAs (HNRNPU-AS1, AC005786.7, and LINC00861) and 3 downregulated circRNAs (RP11-443B7.3, CTD-2616J11.14, and CTD-2616J11.3) were randomly selected for qRT-PCR validation. The results were in accordance with the differential expression observed in RNA-seq.

GO enrichment analysis showed that the differently expressed lncRNAs were mainly associated with metabolic process, catabolic process, and biosynthetic process in biological process; nuclear lumen, organelle lumen, and cytoplasm in cellular component; and protein binding, RNA binding, and molecular function in molecular function. Furthermore, enriched pathways demonstrated that most enriched pathways were related to the calcium signaling pathway, NF-kappa B signaling pathway, cytokine-cytokine receptor interaction, and Toll-like receptor signaling pathway. Accumulating evidence illustrates that atrial tachyarrhythmia causes atrial hypertrophy by activation of the calcium signaling pathway, which thereby contributes to structural remodeling of the human atria [16]. Reports showed that the NF-kappa B signaling pathway and Toll-like receptor signaling pathway are closely associated with apoptosis, immunity, inflammation, and oxidative stress [17, 18]. All these demonstrated that these signaling pathways might be involved in the initiation and development of AF and differentially expressed lncRNAs may participate in the pathogenesis of AF.

In the current study, a coexpression network analysis between lncRNA and mRNA was constructed to predict the key lncRNAs that related to AF by Cytoscape 3.01. HNRNPU-AS1, which was shown to have the highest correlated degree, may play an important role in the coexpression network. The results indicate that the regulation of HNRNPU-AS1 may affect the onset, progression, and maintenance of AF through regulating the expression of their corresponding mRNAs.

In summary, our results illustrated a profile of lncRNAs differentially expressed in monocytes of AF patients and differentially expressed lncRNAs may play core roles in the mechanism of AF. Although the mechanisms of the discovered lncRNAs in AF remain to be elucidated, we hope our novel discovery will lead to more studies that will determine its function.

## Figures and Tables

**Table 1:** Clinical characteristics of AF patients and matched controls.

Parameters	AF patients (*n* = 20)	Controls (*n* = 20)	*t*/*x*^2^	*P* value
Age (years)	64.15 ± 6.65	63.65 ± 5.99	0.25	0.821
Male/female	10/10	12/8	0.40	0.583
Body mass index (kg/m^2^)	23.96 ± 1.98	23.38 ± 1.62	1.01	0.315
Smoking	2	2	0	—
Alcohol abuse	0	0	0	—
NYHA class I/II/III/IV	20/0/0/0	20/0/0/0	0	—
SBP (mmHg)	130.75 ± 15.38	129.35 ± 14.57	0.29	0.782
DBP (mmHg)	73.45 ± 8.54	72.40 ± 8.21	0.39	0.694
LAD (cm)	4.06 ± 0.42	3.24 ± 0.25	7.45	0.008
LVEF (%)	54.92 ± 5.21	56.37 ± 6.28	0.79	0.434

AF: atrial fibrillation; SBP: systolic blood pressure; DBP: diastolic blood pressure; NYHA: New York Heart Association; LAD: left atrium diameter; LVEF: left ventricular ejection fraction.

**Table 2:** The primer sequences for qRT-PCR.

Gene_id	Gene_name	Primer sequences	Length
ENSG00000188206.6	HNRNPU-AS1	F: 5′-GGAAGCTGTACACTGGAGGT-3′	182
		R: 5′-GCGCTAGCACACTGCAATTA-3′	
ENSG00000267436.1	AC005786.7	F: 5′-CAGCAGAGTCCACCAAGC-3′	168
		R: 5′-TGAGCTCAGTCCAGTTCACC-3′	
ENSG00000245164.6	LINC00861	F: 5′-GCCATTCTTCAAGGACTTCACA-3′	112
		R: 5′-CAGCTCCAATTTCCAATTCTGC-3′	
ENSG00000258082.1	RP11-443B7.3	F: 5′-TCACTAGTGTGCCGTCTGAA-3′	154
		R: 5′-GTCGGAACACAGAACACCTG-3′	
ENSG00000268889.1	CTD-2616J11.14	F: 5′-AGCAACTATCTTGGCAACATCCT-3′	105
		R: 5′-AACAACCCTACTTAACGAAACCC-3′	
ENSG00000254760.1	CTD-2616J11.3	F: 5′-TTGGAGAATGCCGTTGAGATG-3′	113
		R: 5′-GCAAAGAGTAGGGTCCTGTGGT-3′	
GAPDH-F		F: 5′-CATGAGAAGTATGACAACAGCCT-3′	177
GAPDH-R		R: 5′-AGTCCTTCCACGATACCAAAGT-3′	

**Table 3:** Differentially expressed lncRNAs.

Differentially expressed	Gene_id	Gene_name	AF_FPKM	C_FPKM	Fold change	*P* value
Upregulated	ENSG00000188206.6	HNRNPU-AS1	8.68	0.72	12.06	0.015
	ENSG00000247982.6	LINC00926	25.85	12.33	2.08	0.043
	ENSG00000245164.6	LINC00861	23.56	3.183	7.21	0.003
	ENSG00000267436.1	AC005786.7	1.91	0.11	18.63	0.002
	ENSG00000271895.2	RP4-635E18.8	9.84	0.23	42.7	0.001
	XLOC_099061	—	2.05	0.18	3.48	0.023

Downregulated	ENSG00000230470.1	GS1-115G20.1	1.23	3.43	2.78	0.033
	ENSG00000241163.7	LINC00877	2.22	4.65	2.11	0.035
	ENSG00000239794		3.72	8.68	2.34	0.032
	ENSG00000175611.11	LINC00476	20.42	49.47	2.47	0.039
	ENSG00000254760.1	CTD-2616J11.3	2.05	6.61	3.31	0.013
	ENSG00000255441.1	CTD-2616J11.2	2.46	7.51	3.12	0.026
	ENSG00000258082.1	RP11-443B7.3	0.74	2.63	3.77	0.002
	ENSG00000261222.2	CTD-2006K23.1	2.12	5.66	2.66	0.002
	ENSG00000268889.1	CTD-2616J11.14	2.02	6.55	3.28	0.012
	ENSG00000271614.1	LINC00936	2.72	8.18	3.02	0.002
	ENSG00000279491.1	RP11-810P12.7	2.11	4.33	2.06	0.042
	XLOC_060065	—	0.86	2.73	3.41	0.046
	XLOC_109634	—	0.22	2.12	9.64	0.001

## Data Availability

The data used to support the findings of this study are available from the corresponding author upon request.
